# Surgical Management of Complicated Abdominal Tuberculosis: The First Systematic Review—New Treatments for an Ancient Disease and the State of the Art

**DOI:** 10.3390/jcm13164894

**Published:** 2024-08-19

**Authors:** Giuseppe Di Buono, Giorgio Romano, Giuseppe Amato, Gabriele Barletta, Giorgio Romano, Nicoletta Adelfio, Girolamo Geraci, Antonino Agrusa

**Affiliations:** Department of Precision Medicine in Medical, Surgical and Critical Care (Me.Pre.C.C.), University of Palermo, 90133 Palermo, Italy; giorgio.romano@unipa.it (G.R.); amatomed@gmail.com (G.A.); gabrielebarletta@gmail.com (G.B.); giorgioromano95@gmail.com (G.R.); nicoletta.adelfio@gmail.com (N.A.); girolamo.geraci@unipa.it (G.G.); antonino.agrusa@unipa.it (A.A.)

**Keywords:** systematic review, tuberculosis, abdominal tuberculosis, laparoscopy, abdominal tuberculosis diagnosis, abdominal tuberculosis treatment

## Abstract

**Background**: Abdominal tuberculosis comprises all forms of tuberculosis that involve the gastrointestinal tract. Controversies exist regarding the surgical approach and timing and type of intervention for complicated forms of abdominal tuberculosis. The aim of this systematic review is to define the rate of surgical treatment, the type of surgical procedures performed and the role of minimally invasive surgery in the management of abdominal tuberculosis. **Methods**: The literature in MEDLINE, Scopus and Google Scholar and forward and backward citations for studies published between database inception and July 2022 were searched without language restrictions. All prospective and retrospective studies were included. The electronic database search yielded 2440 records. Additionally, eight records were identified through snowball searching. Following duplicate removal (45 duplicates found), 2403 records were screened for titles and abstracts. After screening for titles and abstracts and exclusion criteria, 38 reports were included for systematic review, 27 retrospective studies and 11 prospective studies. Data extracted included the general and demographic characteristics of the studies, diagnostic methods used, clinical presentation, site of involvement and details on surgical treatment. **Results**: In total, 2870 patients with a diagnosis of abdominal tuberculosis were included, and 1803 (63%) underwent a surgical procedure. The majority of patients underwent an open surgical procedure (95%). The most commonly performed procedures were adhesiolysis (21%) and small bowel resection with primary anastomosis (21%). **Conclusions**: The results of this review suggest that whenever surgery is required, there is a tendency to perform open surgical procedures in patients with complicated abdominal tuberculosis, both in emergency and elective settings, despite advances in minimally invasive surgery. The study protocol was registered on PROSPERO (CRD42022354322).

## 1. Introduction

**Figure 1:**
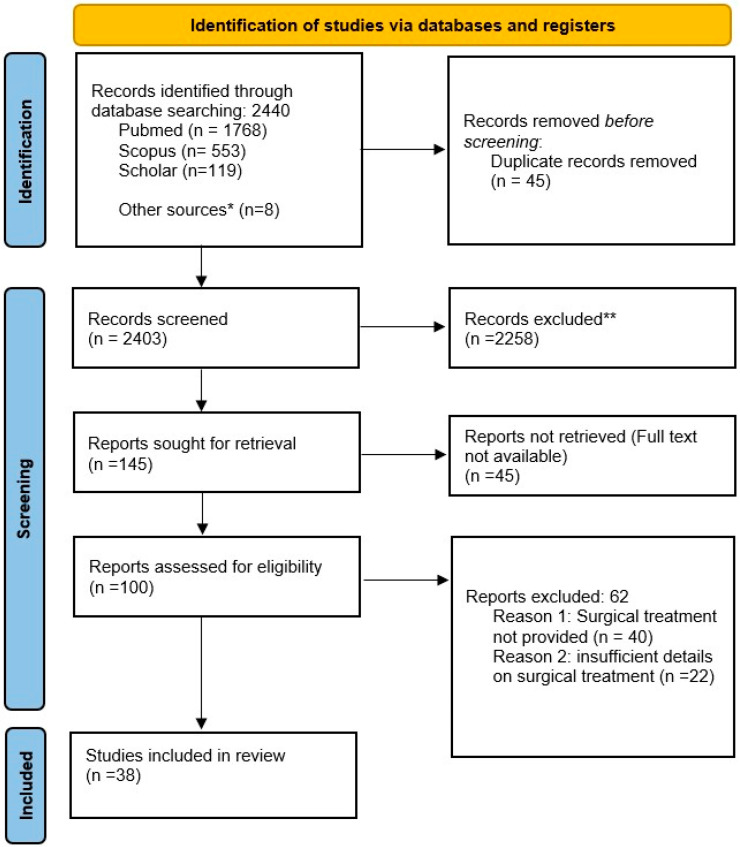
PRISMA 2020 flow diagram of this study. * Other source (n = 8): records identified through snowball searching; ** All the records were excluded by humans for the following reasons: EPTB (extra pulmonary tuberculosis) without abdominal involvement (n = 254); PT (pulmonary tuberculosis) without abdominal involvement (n = 105); Wrong population (children or <16 years) (n = 159); Wrong topic (n = 1116); Wrong study design (n = 71); Case reports and case series (n = 384); Continuous ambulatory peritoneal dialysis patients (n = 66); Transplant patients (n = 37); Articles published previously than 1970 (n = 66).

Tuberculosis (TB) is an infectious disease caused by *Mycobacterium tuberculosis* and until the coronavirus (COVID-19) pandemic, TB was the leading cause of death from a single infectious agent, ranking above HIV/AIDS, with 1.3 million estimated deaths classified as caused by TB in 2020 [1]. Due to its severe social and economic implications, the prevention and treatment of TB have always been a permanent challenge over the course of human history and have recently become more arduous because of the recent appearance of multi-drug-resistant forms [1]. TB is a multisystem disease that can affect any organ of the body [2], with the most commonly reported sites being the lungs (wherein it is termed pulmonary TB), lymphatic system, skeletal system and pleura [3,4,5,6,7,8], followed by the urinary system, digestive system, cerebrospinal meninges and breast [9]. Abdominal TB, whose reports date back to 17th century [2] comprises all forms of TB that involve the gastrointestinal tract, from the mouth to anus (49%), peritoneum (42%), mesenteric lymph nodes (4%) and the solid viscera, including the liver and pancreatic biliary system (5%) [10]. Abdominal tuberculosis, comprising around 5% of all cases of TB worldwide, is the sixth most prevalent presentation of extrapulmonary tuberculosis in Europe [10]. The relevance of this rare manifestation of TB has recently increased due to the influx of immigration and prevalence of HIV infection, which are the main risk factors for development of abdominal TB [2]. The prognosis remains poor in cases of late starting of anti-tubercular therapy, as happens in cases of negative diagnostic work-up. In those cases, surgery is required for diagnosis even in cases of uncomplicated abdominal TB. Controversies exist regarding the surgical approach and timing and type of interventions for acute complicated cases of abdominal tuberculosis, such as bowel perforation, acute bowel obstruction and complicated chronic cases, including chronic bowel obstruction, fistula, tubercular abscess and abdominal masses [3,6] Since its introduction, laparoscopy has emerged as a safe and rapid investigation tool for the direct visualization of the peritoneal cavity in cases of suspected abdominal tuberculosis [7,8], and more recently, its effectiveness and feasibility have been reported in treatment [9]. The scientific literature on this theme is extremely varied and mostly represented by case reports, and no guidelines exist regarding the surgical management of abdominal tuberculosis. In light of the above, it is important to provide a guide on surgical treatment modalities in order to trace initial steps for better clinical practice and future guidelines. The following systematic review was conducted and reported in line with the PRISMA 2020 guidelines (Figure 1).

### Aims

The aims of the study are (1) to understand how to define the rate of surgical treatment among patients with a diagnosis of abdominal tuberculosis in order to evaluate the role of surgery in the treatment of abdominal tuberculosis, (2) define the type of surgery performed, (3) define the role of minimally invasive surgery in the treatment of abdominal TB, and (4) trace initial steps for future guidelines for the treatment of abdominal tuberculosis and creation of an international surgery registry.

## 2. Materials and Methods

### 2.1. Search Strategy 

The literature in the MEDLINE (through PubMed) database, Scopus and Google Scholar was searched from its detection to July 2022. Despite the fact that reports of surgical treatment of abdominal TB have been published since the early years of the twentieth century [10], for the purpose of this review, we chose to screen articles published after 1970 in order to obtain reliable sources in line with modern surgical advances. The following search strategy was used for PubMed searching: (“Tuberculosis, Gastrointestinal”[Mesh] OR “Peritonitis, Tuberculosis”[Mesh] OR “extrapulmonary tuberculosis” OR “abdominal tuberculosis” OR “Tuberculous peritonitis”) AND (“Intestinal Perforation”[Mesh] OR “Intestinal Obstruction”[Mesh] OR “Abdomen, Acute”[Mesh]). Additionally, reference lists of various papers and books were screened.

### 2.2. Selection Process

All the identified records were de-duplicated by an author using Rayyan (http://rayyan.qcri.org (accessed on 1 August 2022)), followed by manual search. After de-duplication, the full text of the selected studies was retrieved and independently assessed for eligibility by two authors, and any disagreement was resolved by discussion with a third author.

### 2.3. Inclusion and Exclusion Criteria

Inclusion criteria were adult patients (aged superior of 18 years) with a definitive diagnosis of abdominal tuberculosis (pre or postoperative) who received both non-surgical and surgical treatment, both in emergency and elective settings. Exclusion criteria were patients aged under 18 years old and patients with extrapulmonary tuberculosis without abdominal involvement. Transplanted patients, cirrhotic patients and patients on continuous ambulatory peritoneal dialysis who developed tubercular peritonitis were also excluded since they represented patients for whom only non-surgical treatment was conceivable, departing from the review’s aim. Articles written in languages other than English were translated with an automatic translator. Articles that did not provide separate details for the patients with abdominal tuberculosis and other forms of extrapulmonary tuberculosis were excluded due to the impossibility of extracting data for cumulative analysis. Initially, all studies were screened, including prospective and retrospective studies, case series and case reports. Subsequently, in order to evaluate the reported rate of surgical treatment among patients with abdominal TB, only prospective and retrospective articles were considered for data synthesis, whereas case reports and case series were excluded but were considered to develop discussion and outline the newest trends in the treatment of abdominal tuberculosis. The diagnosis of abdominal tuberculosis was considered definitive either in the case of the culture of tissue (e.g., intestinal and peritoneum biopsy, lymph nodes) or ascitic fluid positive for *Mycobacterium tuberculosis* or the histological demonstration of typical acid-fast bacilli (AFB) or histological evidence of caseating granuloma or TB polymerase chain reaction (PCR) performed on a biopsy specimen [10,11,12,13].

### 2.4. Data Synthesis and Analysis 

Data extracted included the author, the year, the country, the number of clinical cases included and number of patients who underwent surgery, demographic information (age, gender), the setting (emergency, elective or both), diagnostic methods for abdominal tuberculosis (laboratory findings, immunoassay, ultrasound and CT scan of abdomen, colonoscopy, diagnostic laparoscopy and laparotomy, histopathological evidence of caseating granuloma and/or acid-fast bacilli, microbiological culture, polymerase chain reaction—PCR), clinical presentation (abdominal distension, abdominal pain, fever, vomit, nausea, weight loss, night sweats, peritoneal signs, intestinal obstruction, abdominal mass), onset (acute, subacute and/or chronic), an abdominal tuberculosis-specific diagnosis (bowel perforation, bowel obstruction, tubercular peritonitis, tubercular lymphadenopathy, abdominal cocoon, tubercular abscess, strictures), the site of involvement, the surgical approach (open or laparoscopic), conversion to open surgery (in case of laparoscopic initial approach) and details on the surgery performed (adhesiolysis, stricturoplasty, biopsy, appendectomy, lymphadenectomy, small bowel resection without primary anastomosis, small bowel resection with primary anastomosis, direct suture, hemicolectomy). Risk of bias was not assessed formally because most of the articles included were retrospective and prospective studies. Meta-analysis could not be undertaken due to the heterogeneity of interventions, settings, study designs and outcome measures. The study protocol was registered on PROSPERO (CRD42022354322) and is available via the following link: https://www.crd.york.ac.uk/prospero/display_record.php?RecordID=354322 (accessed on 18 August 2022).

## 3. Results

The electronic database search yielded 2440 records. Additionally, eight records were identified through snowball searching. Following duplicate removal (45 duplicates found), 2403 records were screened for titles and abstracts. After screening for titles and abstracts, 145 reports were sought for retrieval, whereas 2258 were excluded. Records were excluded for the following reasons:

- Extra pulmonary tuberculosis (EPTB) without abdominal involvement (n = 254);
- Pulmonary tuberculosis (PT) without abdominal involvement (n = 105);
- Wrong population (children or <16 years) (n = 159);
- Wrong topic (n = 1116);
- Wrong study design (n = 71);
- Case reports and case series (n = 384);
- Continuous ambulatory peritoneal dialysis patients (n = 66);
- Transplant patients (n = 37);
- Articles published earlier than 1970 (n = 66).

A total of 45 reports were not retrieved; therefore, only 100 reports were assessed for eligibility.

A total of 38 reports were included in the review, and 62 reports were excluded for the following reasons:

- Reason 1: Surgical treatment not provided (n = 40);
- Reason 2: insufficient details on surgical treatment (n = 22).

### 3.1. Geographic Distribution (Figure 2a)

**Figure 2:**
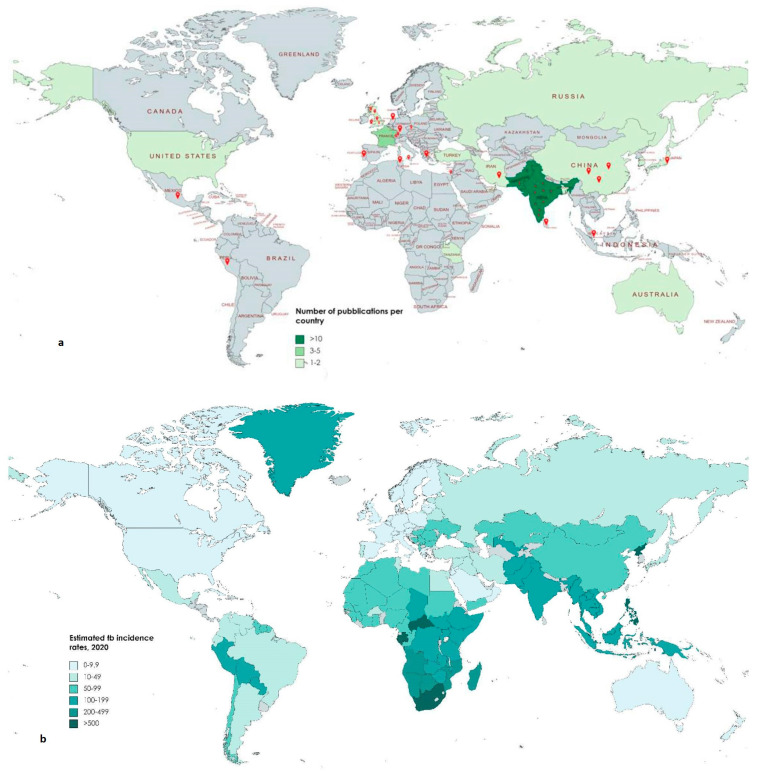
Geographic Distribution.

Most publications focused on Asian case reports and longitudinal studies [14,15,16,17,18], followed by Europe [19], North America and Africa. Some countries dominate the published studies within their respective region, such as India (21 publications, which is the highest number of publications of a single country) in Asia, the UK in Europe (6 publications), and USA in the North America (5 publications). These results are partly in contrast with worldwide estimated TB incidence rates, as shown in Figure 2b.

### 3.2. Demographic and Clinical Data 

In grouping the included articles by publication type, 27 retrospective studies and 11 prospective studies were included. The years of publication of the included studies ranged from 1977 to 2021, with 2014 to 2016 producing the most publications. Table 1 provides the general characteristics and demographic data of the reviewed studies, including the year of publication, type of publication and country. The articles comprised 2971 patients with a diagnosis of abdominal tuberculosis and 1803 (60.7%) patients who received surgical treatment, but only in 1014 patients did the studies report the surgical approach used. The demographic data of the patients are summarized on Table 1. Among included studies, 5 focused exclusively on the emergency treatment of abdominal tuberculosis, whereas 27 study included both emergency and elective procedures, 1 study included only on elective procedures and 5 studies did not reported the setting. There was a slight predominance of male patients (n. 1577, 55%) compared to women (n. 1394, 49%).

### 3.3. Diagnostic Evaluation

Twenty-eight studies reported the use of imaging test for preoperative assessment. Eighteen studies reported the use of abdominal ultrasound (US) [11,12,13,14,15,16,17,18,19,20,21,22,23,24,25,26], but in most cases, it resulted in nonspecific outcomes. The use of abdominal CT was reported in 17 of the 38 studies included. Common imaging findings were bowel loop thickening, ascites and lymphadenopathy. Endoscopic exams, such as colonoscopy, were performed in seven studies [13,18,24,25,26,27,28] and resulted in nonspecific outcomes (four cases of colonic ulcer, two cases of colonic masses). In contrast, intraoperative findings during laparoscopy were reported in eleven papers as extremely useful for reaching a diagnosis [12,15,21,22,26,28,29,30,31]. Twenty-five studies [12,15,16,17,18,20,21,22,24,25,26,28,32,33,34,35,36,37,38,39,40,41] reported the use of histopathological examination for definitive diagnosis through evidence of caseating granuloma or acid-fast bacilli. PCR, of both blood and ascites, was used only in six studies [15,16,22,23], although it has been demonstrated that it can be used as an effective tool to diagnose abdominal TB, and the costs of the procedure do not appear to be a limit of its use [16,42,43].

### 3.4. Clinical Presentation

Clinical symptoms reported included abdominal pain (n. 2306), followed by vomiting (n. 1510), fever (n. 1471), weight loss (n. 1259), abdominal distension (n. 865), nausea (n. 1259), diarrhea (n. 461) and constipation (n. 749). Other common symptoms were diarrhea, night sweats and peritoneal signs (Table 2). The most frequent diagnosis was bowel obstruction (20%, n. 563), followed by bowel perforation (14%, n. 410), tubercular peritonitis (13%, n. 378) and tubercular lymphadenopathy (8%, n. 223). Other diagnoses reported included abdominal masses (9%, n. 259), abdominal cocoon (1%, n. 43), tubercular abscess (0.38%, n. 11) and strictures (6%, n. 173) (Table 3). The most commonly involved sites were the ileum (25%, n. 730), lymph nodes (13%, n. 383), the peritoneum (15%, n. 433), the jejunum (4%, n. 111), the appendix (1%, n. 30), the colon (3%, n. 90), the gallbladder (0.28%, n. 1), the spleen and the liver (2%, n. 59) (Table 4). Concomitant pulmonary tuberculosis was described only in 430 (15%) patients

### 3.5. Surgical Approach and Treatment

Twelve studies did not report the surgical approach used. Therefore, only twenty-six studies, with a total of 1014 patients, reported the surgical approach used: the majority of patients underwent open surgery (n. 963, 95%), while only 51 (5%) patients underwent a laparoscopic procedure. Of these, 16 diagnostic laparoscopies were reported and 3 (5.88%) cases of conversion occurred. Overall, the most commonly performed surgeries were adhesiolysis (n. 373, 21%) and small bowel resection with primary anastomosis (n. 372, 21%), followed by small bowel resection without primary anastomosis (n. 314, 17%), direct sutures (n. 191, 11%), hemicolectomy (n. 202, 11%), stricturoplasty (n. 186, 10%), biopsy (n. 130, 7%), appendectomy (n. 32, 6%) and lymphadenectomy (n. 21, 1%) (Table 5).

## 4. Discussion

Abdominal tuberculosis is an increasingly common manifestation of Tuberculosis, mainly due to the higher influx of immigrants and HIV infections. It poses significant diagnostic challenges, since its manifestations, both acute and chronic, mimic different diseases [2,5]. 

### 4.1. Diagnostic Evaluation

The diagnostic work-up for abdominal tuberculosis includes imaging-based, laboratory and pathological examination. In this review’s results, imaging exams, such as CT scan and abdominal ultrasound, showed bowel loop thickening, ascites and lymphadenopathy. These results are nonspecific; nevertheless, they were used in almost all studies, in accordance with the existing literature that recommends including radiological imaging in the work-up for abdominal tuberculosis [47,48,49,50]: ultrasound is safe, portable, inexpensive and non-invasive [51], but CT shows higher sensitivity and specificity [52] and is also becoming available and affordable in resource-limited settings, as shown in the article of Singh et al. [26]. With regard to endoscopic exams, they are useful for differentiating abdominal TB from other diseases, especially Crohn’s disease. In this review, colonoscopy and subsequent histologic examination had diagnostic results only in two cases [15], and this result could be explained by the low prevalence of colonic involvement of abdominal TB (reported in 13 patients, 1%). Laboratory exams, such as immunoassay and PCR, are considered useful for reaching diagnosis, and several studies on their effectiveness have been published [16,42,43]. Determining the actual rate of preoperative diagnosis of tuberculosis is complex as the diagnosis of suspicion is based on several factors including familiarity with the disease (especially in areas of low prevalence) and access to appropriate preoperative investigations. The literature shows that the preoperative diagnosis of tuberculosis occurs in between 20% and 53% of patients. We can improve the preoperative diagnosis of abdominal tuberculosis with the use of appropriate radiological examinations. The most used are barium meal, ultrasound and CT scans. Common features suggestive of abdominal tuberculosis on barium X-rays are luminal narrowing with the proximal dilation of bowel loops. On CT scan, the presence of ascites, enlarged lymph nodes and, in particular, omental thickening and bowel wall thickness are the typical findings. Among these methods, CT abdominal scans have the most specificity, although imaging could mimic neoplastic pathology with peritoneal carcinomatosis. Really, no single diagnostic method per se is adequate for the preoperative diagnosis of abdominal tuberculosis in all patients.

### 4.2. Clinical Presentation and Management

Early recognition of abdominal TB is essential to promptly start medical treatment and reduce morbidity and mortality, whose rates are high in untreated cases. Despite the fact that the majority forms of abdominal tuberculosis could be managed through anti-tubercular therapy, several complications can occur, including intestinal obstruction, perforation, fistulae and abscesses [52,53,54,55,56]. As Table 2 and Table 3 show well, the symptoms and clinical presentation of abdominal tuberculosis can be nonspecific and multiple in nature. In daily practice, several clinical features may lead to the suspicion of abdominal TB: previous history of tuberculosis, any anti-tubercular therapy, concomitant HIV infection, radiological evidence of pulmonary tuberculosis, malnutrition and a history of previous abdominal surgery. In the absence of a strong preoperative diagnostic suspicion of abdominal tuberculosis, intraoperative evaluation may be very difficult. Certain intraoperative elements may guide the surgeon towards the diagnosis of abdominal tuberculosis: miliary tuberculosis, peritoneal and intestinal thickening, omental cake, multiple dense adhesions and intestinal fistula. But all these findings may simulate peritoneal carcinomatosis or inflammatory bowel disease. The primary aim of this review was to evaluate the role of surgery for abdominal TB. In accordance with the different authors (Table 3), we consider complicated abdominal TB cases to be those represented by bowel obstruction (20%), bowel perforation (14%) and tubercular peritonitis (13%) in which an emergency surgical approach is definitely required. The other clinical presentations generally managed in an integrated medical and surgical manner, depending on the characteristics of the individual patient. The results highlighted the existence of a controversial rate of surgical treatment: most of the retrospective studies that included patients managed through both medical and surgical treatment reported that the majority of patients with complicated abdominal TB, both in emergency and elective settings, required surgical treatment [2,3,4,5,6,7]. However, in the retrospective study of Chaudhary et al. [35], only 36% of patients received surgical treatment, whereas the remaining were treated conservatively. The design of this study, a single-center retrospective study based on two decades of experience in a high-incidence country (India), makes it a reliable source to analyze clinical presentation, diagnostic evaluation, treatment modalities and outcomes. However, the results must be weighed up since they excluded HIV-co-infected patients and included both in- and out-patients with various clinical presentations. Indeed, patients were divided into four groups based on the clinical presentation, including patients with chronic abdominal pain, patients with ascites, patients with abdominal mass and patients with acute pain abdomen. So, we can see that in this classification, only patients with acute abdominal pain can be considered to have complicated abdominal TB. The results of Chaudhary et al. [35] were related to this classification. In fact, among these groups, surgery was needed in 209 patients (58.3%) with acute abdominal pain, and it was required only for 16 patients (6.7%) with chronic abdominal pain and 14 patients (20%) with abdominal mass to obtain diagnosis. Similar results were obtained by the retrospective study of Z. Wang et al. [23], which included 108 patients with clinical and radiological diagnoses with various types of abdominal tuberculosis (lymphatic type, intestinal type, peritoneum type and mixed type). All the patients included were given a standard first-line anti-tuberculosis regimen, and surgery was performed in cases of free perforation, confined perforation with abscess or fistula, massive bleeding, complete obstruction and obstruction unresponsive to medical management. Interestingly, the study found the highest rate of surgical treatment among patients with the lymphatic type (70%) and mixed type (68.2%) of tuberculosis, with lower figures for the intestinal (50%) and peritoneal types (8.3%). However, the rate of surgical treatment was still high for the intestinal type of abdominal tuberculosis, and this could be due to the presence of intestinal tuberculous granulomas and necrotic tissue that lead to blockages in the intestinal lumen, as well as to the presence of areas of fibrotic stenosis that are difficult to effectively relieve by anti-tuberculosis treatment. Moreover, the results of this study suggested that even though patients with some types of peritoneal abdominal tuberculosis had symptoms of incomplete obstruction, this type of abdominal tuberculosis was the one that responded more favorably to medical treatment with no recurrence of the obstructions. Another important study that was reviewed to evaluate surgical treatment rate was the prospective study of Wani et al. [16], which reported that 76% of patients required emergency surgical procedures. In detail, although this percentage differs from the abovementioned figure, it could be explained by considering the fact that conservative treatment was reserved for patients with features of subacute intestinal obstruction, reporting a dramatical improvement as soon as they were put on anti-tubercular chemotherapy, whereas surgery was required in emergencies for acute intestinal obstruction, gut perforation and peritonitis, as well as in an elective setting for those patients in whom conservative treatment failed and gut obstruction was present. Unfortunately, the article did not provide details regarding the percentage of patients who needed surgical treatment after initial conservative therapy. The percentage reported is similar to that of another prospective study, the one of Chalya et al. [14]. Even though the setting is different (a tertiary-care hospital of a high prevalence and poorly resourced country—Tanzania), patient follow up was similar and suggests that when considering all patients presenting with a clinical diagnosis of abdominal tuberculosis, with both acute and chronic symptoms, treatment is primarily conservative but surgery is needed for complicated cases, in which emergency exploratory laparotomy is reported, as well as in unresponsive cases. One of the limits of this review is, therefore, the absence of homogeneity of the studies included with regard to the inclusion criteria, since some studies included only patients who received surgical treatment, whereas others included all patients with a diagnosis of abdominal tuberculosis who both received surgical and non-surgical treatment, making comparison of treatment efficacy and evaluation of the surgical treatment rate difficult and biased. Furthermore, this review aimed to question the role of laparoscopy in therapeutic intervention for abdominal tuberculosis. To this end, the use of laparoscopy, both for diagnosis and for treatment, was evaluated. In this review, ten articles reported the use of laparoscopy. In the article of Mousa et al. [13], a laparoscopic approach was used in five patients: in one case, it was used for diagnostic purposes, as one patient underwent elective laparoscopic surgery (cholecystectomy), while three patients were converted and underwent hemicolectomy and adhesiolysis for abdominal cocoon. However, there are no details available regarding the clinical presentation of those patients who underwent initial laparoscopic treatment and were eventually converted to an open approach or any information regarding intraoperative findings in those cases. The article of Singh et al. [26] reported that three patients had laparoscopic resections: two via right hemicolectomy and one via ileocecal resection. There are no details regarding the surgical setting and intraoperative findings or the extent of the disease that led to surgical procedures being used. The article of Fillion et al. [34] reported that laparotomy was used in all cases of complicated abdominal tuberculosis (intestinal obstruction and perforation), while in the absence of complications, laparoscopy was used to establish diagnosis and obtain tissue samples. Diagnostic laparoscopies were reported in four further studies including the study of Hassan et al. [46], where eight patients underwent diagnostic laparoscopy and laparoscopic peritoneal biopsy, whereas nine patients underwent open surgical procedures: laparotomy was performed for complications of abdominal tuberculosis in six patients and to obtain a tissue diagnosis in three patients. Hence, the results of this review showed that the most used surgical approach was the open approach, both in elective and emergency surgery. It should be noted that the current literature review has evidenced that even in an industrialized country such as the United States, where it is plausible to believe that there is access to considerable economic and technological resources even in tertiary care hospitals, laparoscopy has played a marginal role in the treatment of abdominal tuberculosis, as reported in the study by Hassan et al. [46]. Interestingly, although all surgeries except one (tubo-ovarian abscess) were performed in elective settings, an open approach was used for procedures that could have been managed through a laparoscopic approach, such as gastrojejunostomy, gastrostomy, jejunostomy, right hemicolectomy with primary anastomosis and excisional biopsies. These findings, such as the ones of the study of Fillion et al. [34], reveal that even if laparoscopy was extremely useful for reaching a diagnosis, in accordance with the existing literature [7], especially when non-invasive diagnostic work-up was negative, its use is not established for the treatment of this disease. Differences in the socioeconomic levels of countries do not explain this reticence, since the reported rate is similar between the USA, France (a highly developed country with a relatively low incidence of abdominal TB) and India (a high-prevalence country). One of the main factors that seemed to limit the use of laparoscopy was, above all, the emergency setting, as well as the fear of finding multiple adhesions that would interfere with the creation of an optimum working space through pneumoperitoneum. Such qualms, however, cannot represent a real obstacle to the use of laparoscopy to date, since there are countless publications demonstrating both the feasibility of using this approach in emergencies, especially for experienced surgeons, without additional risks to the patient and the possibility of performing safe viscerolysis. In this regard, it is worth mentioning that in recent years, several case reports of laparoscopically treated cases of abdominal tuberculosis, even in in low-resource countries, have been published [27,57,58,59,60,61], as effective laparoscopic treatment of emergency complicated cases of abdominal tuberculosis [9], thus indicating an increasing tendency to undertake a surgical approach that is proven to demonstrate benefits in terms of postoperative outcomes.

### 4.3. Type of Surgical Procedures

Finally, the types of surgical procedures performed were evaluated. As shown in Table 3 and Table 5, surgery is required in abdominal emergencies or when surgery is necessary for diagnosis (laparoscopy and/or biopsy). The results of this review indicated that the most common surgical procedures were adhesiolysis (21%) and intestinal resection with primary anastomosis (21%), followed by resection without anastomosis (17%), direct suture of perforated intestinal tract (11%) and hemicolectomies (11%). There were few data available regarding intraoperative findings and the reasons for the choice of direct sutures instead of bowel resection in case of bowel perforation. For instance, the retrospective article of Zhi Wang et al. [23] reported that in case of lymphatic abdominal tuberculosis, the severity of the adhesive intestinal obstruction, secondary to the rupture of necrotic tuberculous lymph nodes, led, in most cases, to doctors performing adhesion lysis, with or without enterostomy, whereas in the case of intestinal tuberculosis, wherein adhesions are mainly located in the proximity of the lesion area, the most commonly performed surgical procedure was intestinal resection. Despite this study providing details on type of surgical procedure performed according to the clinical, anatomical and intraoperative characteristics of abdominal tuberculosis, further data from prospective and larger studies are required in order to guide surgical treatment. The results of this review suggest that, given the pleomorphic presentation of the disease, there is not a standard surgical procedure, and the choice of surgical procedure, therefore, may vary depending on the site and the extent of disease, nutritional and general condition of the patient, expertise available, local protocols and surgeon’s preference. In this regard, it could be beneficial to use a laparoscopic approach even in cases of suspected abdominal cocoon, since it allows the visualization of the characteristic encasement of the bowel with a thick fibrous membrane and gives the possibility of performing a safe and efficient adhesiolysis with the benefits of a minimally invasive approach. To the best of our knowledge, there are no cases of TB abdominal dissemination after laparoscopic surgery. However, for abdominal tuberculosis, medical treatment is still the main therapy. Surgical treatment is mostly used in the event of emergency complications such as intestinal obstruction and intestinal perforation, as well as when diagnosis is difficult. Abdominal tuberculosis is a series of diseases with multiple manifestations, and the surgical method selected is highly dependent to the surgeon’s experience and judgment and the condition of the hospital. Considering the possibility of relapse of the disease, sparing surgery is recommended (direct sutures were the most commonly reported surgical procedures), despite no official guidelines and protocols existing. From this prospective, a laparoscopic approach could be considered, whenever possible, a conceivable option, combining the intraoperative advantages of pneumoperitoneum (magnification could help with identifying the extent of peritoneal and bowel involvement) with better postoperative outcomes. 

### 4.4. Limitations and Strengths of this Study

The limitations of this study are derived from a further consideration worthy of attention concerning the heterogeneity of published papers regarding the treatment of abdominal tuberculosis, the frequency of which does not fairly reflect the incidence of cases of abdominal tuberculosis (Figure 2). This discrepancy may be due to several reasons that probably reflect the different usability of the scientific literature globally, with it being exceedingly accessible in industrialized countries and extremely limited and lacking in developing countries. This variation inevitably makes the interpretation of the results flawed, so it would be desirable to create a registry of all surgically treated cases of abdominal tuberculosis within which surgeons can spread the type of intervention and outcome obtained, thus promoting the dissemination of scientific evidence. This article collected the studies on abdominal tuberculosis from 1970 to 2021, focusing on the details of the surgical treatment of patients with abdominal tuberculosis and exploring the role of surgical treatment in abdominal tuberculosis, which was a systematic and detailed analysis. This review creates a platform for further research into exploring ways to surgically treat patients with complicated abdominal TB.

## 5. Conclusions

The results of this review, despite the limitations arising from the lack of data and details on the surgical approach, procedures performed, clinical setting and intraoperative findings, outline that surgery plays a limited role in the treatment of uncomplicated forms of abdominal TB, and in some complicated forms, the resolution of symptoms and disease regression can be achieved with prompt anti-tubercular treatment. In contrast, surgery represents the only possible therapy for all acutely complicated forms, especially the ones that involve the gastrointestinal tract. Whenever surgery is required, there is a tendency to perform open surgical procedures in patients with complicated abdominal tuberculosis, both in emergency and elective settings, despite advances in minimally invasive surgery.

### Protocol Amendments

Language restrictions were not applied. In order to achieve the aim of evaluating the rate of surgical treatment among patients with abdominal TB, all the studies that included patients with a definitive diagnosis of abdominal tuberculosis (pre- or postoperative) who received both non-surgical and surgical treatment, both in emergency and elective settings, were included. Further exclusion criteria that were added were transplanted patients, cirrhotic patients and patients on continuous ambulatory peritoneal dialysis who developed tubercular peritonitis. These patients were excluded too since they represented patients for whom, in most cases, only non-surgical treatment was conceivable.

## Figures and Tables

**Table 1:** General characteristics and demographic data of included studies; age was expressed as mean and interquartile range (values in brackets).

Author	Year	Location	Study Design	No_Patients	Patients_Operated	% Patients Operated	Age	Gender_M	Gender_F	Setting
Akgun Y et al. [10]	2004	Turkey	Retrospective and prospective study	80	80	100%	43.9	36	44	both emergency and elective
Yilmazlar et al. [11]	1992	Turkey	retrospective study	17	17	100%	(11–66)	7	10	both emergency and elective
Barot et al. [12]	2021	Mauritius	prospective study	50	50	100%	32.24	26	24	both emergency and elective
Mousa et al. [13]	2021	United Arab Emirates	retrospective study	24	24	100%	(19–59)	11	13	both emergency and elective
Chalya et al. [14]	2013	tanzania	prospective study	256	212	83%	28	148	108	both emergency and elective
Ali et al. [15]	1993	india	retrospective study	9	6	67%	42.1	6	3	both emergency and elective
Wani et al. [16]	2012	India	prospective study	50	38	76%	(10–30)	26	24	both emergency and elective
Kita et al. [17]	1977	Japan	retrospective study	27	7	26%	(25–55)	14	13	elective
Rahman et al. [18]	2014	Bangladesh	retrospective study	50	2	4%	29.3	26	24	both emergency and elective
Klimach et al. [19]	1985	UK	retrospective study	109	76	70%	30.6	52	58	both emergency and elective
Abbasi et al. [20].	2004	Iran	retrospective study	10	10	100%		6	4	emergency
Yousfani et al. [21]	2020	Pakistan	retrospective study	39	39	100%	26.8	22	17	not reported
Ahmad QA et al. [22]	2019	Pakistan	retrospective study	80		0%	26.5	36	44	both emergency and elective
Zhi Wang et al. [23]	2019	china	retrospective study	108	35	32%	(8–81)	63	45	both emergency and elective
Mandavdhare et al. [24]	2019	india	retrospective study	93	8	9%	35.9	53	40	not reported
Beloborodov et al. [25]	2019	russia	retrospective study	165	140	85%	31	127	38	both emergency and elective
Singh et al. [26]	2018	India	retrospective study	35	35	100%	(12–80)	26	9	both emergency and elective
Javed et al. [27]	2017	Pakistan	prospective study	30	30	100%		16	14	not reported
Iqbal et al. [28]	2017	Pakistan	prospective study	40	40	100%	27.5	14	26	emergency
Afridi SP et al. [29]	2016	Pakistan	prospective study	100	96	96%	30	45	55	both emergency and elective
Charokar et al. [30]	2016	India	retrospective study	72	72	100%	30	44	28	both emergency and elective
Pathak et al. [31]	2016	India	prospective study	31	31	100%		17	14	both emergency and elective
Gondal S.H et al. [32]	2016	Pakistan	retrospective study	18	18	100%	(18–35)	11	7	emergency
Cavalli et al. [33]	2016	France	retrospective study	34	4	12%	40	22	12	both emergency and elective
Fillion et al. [34]	2016	France	retrospective study	21	16	76%	51	9	12	both emergency and elective
Chaudhary et al. [35]	2016	India	retrospective study	756	269	36%	(11–20)	410	346	not reported
Awasthi et al. [36]	2015	India	retrospective study	48		0%	27.4	28	20	not reported
Kumar M. et al. [37]	2015	India	prospective study	42	36	86%	(20–39)	20	22	both emergency and elective
Mukhopadhyay et al. [38]	2014	India	prospective study	70	64	91%		27	43	emergency
Kim et al. [39]	2014	South Korea	retrospective study	18	18	100%	38	10	8	emergency
Gill P et al. [40]	2013	Australia	retrospective study	20	11	55%	34	9	11	both emergency and elective
Saaiq et al. [41]	2012	Pakistan	retrospective study	133		0%	28.21	110	123	both emergency and elective
Sadiq et al. [42]	2012	Pakistan	retrospective study	100	83	83%	(25–45)	43	57	both emergency and elective
Imran et al. [43]	2010	Pakistan	prospective study	50	36	72%	31	16	34	both emergency and elective
Arif et al. [44]	2008	Pakistan	retrospective study	50	48	96%	29	20	30	both emergency and elective
Collado et al. [45]	2005	France	retrospective study	17	10	59%	43.9	11	6	both emergency and elective
Hassan et al. [46]	2002	USA	retrospective study	18	17	94%	44	10	8	both emergency and elective

**Table 2:** Clinical presentation: Abd. Distension: abdominal distension; Abd. Pain: abdominal pain; W.L: weight loss; N.S: night sweats; P.S: peritoneal signs; Const.: Constipation; Tendern.: Tenderness; Int. Obstruction: Intestinal obstruction.

Author	Abd. Distension	Abd. Pain	Fever	Vomit	Nausea	W.L	Diarrhea	N.S	P.S	Const.	Ascites	Tendern.	Int. Obstruction
Akgun Y et al. [10]	42	71	49			55						58	11
Yilmazlar et al. [11]	9	14	2	6				2			4	13	5
Barot et al. [12]	19	50	23	30	30	10	7			25			
mousa et al. [13]	9	23	9	5	16	15	13	6	2		8	18	
Chalya et al. [14]	94	240	86	204		122	78		70	165		70	
Ali et al. [15]		6	7	7	7	7							
Wani et al. [16]	22	43	22	28		41	5		10	24	8	38	
Kita et al. [17]													
Rahman et al. [18]	23	43	44	35	35	50				42	21		
Klimach et al. [19]			84	49	40		58	15			9		
Abbasi et al. [20].		9	2			1							
Yousfani et al. [21].		29	72		62	62	46			43	62		
Ahmad QA et al. [22]	29	72		62	62	46	45		43	62			35
Zhi Wang et al. [23]	50	98	58	77	77	45	20			20		35	
Mandavdhare et al. [24]		89	63		49	73	4						30
Beloborodov et al. [25]		146		49	49								
Singh et al. [26]		35	13			21			10		1		24
Javed et al. [27]													
Iqbal et al. [28]													
Afridi SP et al. [29]		72	38			72					42		
Charokar et al. [30]	58	70	31	58	58	18	27		37	27		57	
Pathak et al. [31]	6	31		18	18					4			
Gondal S.H et al. [32]													
Cavalli et al. [33]		24	24	12		16	4	4			19		
Fillion et al. [34]		14	12			16	3			3	7		
Chaudhary et al. [35]	481	756	496	686	586	481	121		242	236	121		
Awasthi et al. [36]		48	42	12	12								
Kumar M et al. [37]		15	38	18	25							25	3
Mukhopadhyay et al. [38]		70	70								7	61	
Kim et al. [39]		18	7		14		5	9		2			4
Gill P et al. [40]	7	13	7	5		6		5	3	1	7	11	3
Saaiq et al. [41]													47
Sadiq et al. [42]		82	80	65	65				35	70			51
Imran et al. [43]	12	50	45	40	40		25			25			
Arif et al. [44]		47	31	30					12				
Collado et al. [45]		15	10	9	9	15							5
Hassan et al. [46]	4	13	6	5	5	11		3				3	
	865	2306	1471	1510	1259	1183	461	44	464	749	316	389	218
	29%	77%	49%	51%	42%	40%	15%	1.5%	16%	25%	12%	13%	7.3%

**Table 3:** Preoperative diagnosis in patients with abdominal tuberculosis.

Author	Bowel Perforation	Bowel Obstruction	Peritoneal_Tuberculosis	Tubercular Lymphadenopathy	Abdominal Cocoon	Tubercular Abscess	Acute_Appendicitis	Abdominal_Mass	Strictures	Ascitis
Akgun Y et al. [10]	9	11								
Yilmazlar et al. [11]	3	8	1					2		
Barot et al. [12]	15							10		
mousa et al. [13]		1	1			1	4	3	.	
Chalya et al. [14]	32	127	106	10				17	78	
Ali et al. [15]		5						1		
Wani et al. [16].	3			16	2				5	
Kita et al. [17]										
Rahman et al. [18]	2	2								
Klimach et al. [19].		13				2	17			5
Abbasi et al. [20].	2	2	5			2		2		
Yousfani et al. [21]	25	12	27		1					
Ahmad QA et al. [22]		80						19		
Zhi Wang et al. [23]	9		82	30		6		9		
Mandavdhare et al. [24]								10		
Beloborodov et al. [25]	75		22	16			9			
Singh et al. [26].	10	23	16	28	4			7	17	
Javed et al. [27]	6	24								
Iqbal et al. [28]	17	15		4	15			3	5	
Afridi SP et al. [29]		24	61					7		
Charokar et al. [30]	18	29			6			25	6	
Pathak et al. [31]	9	20					1		.	
Gondal S.H et al. [32]	7	4							7	
Cavalli et al. [33]	2		2					1		
Fillion et al. [34]	1	1	8					2	1	
Chaudhary et al. [35]	78	52	39	112	8			77	52	
Awasthi et al. [36]	31	42								
Magesh Kumar et al. [37]	2	3						9	2	
Mukhopadhyay et al. [38]	22	33			3		7	3		5
Kim et al. [39]	18								.	
Gill P et al. [40]								3		
Saaiq et al. [41]					4					
Sadiq et al. [42]								10		
Imran et al. [43]								30		
Arif et al. [44]	14	25						2		
Collado et al. [45]		7						4		
Hassan et al. [46]			8	7				3	.	
	410	563	378	223	43	11	38	259	173	10
	14%	20%	13%	8%	1%	0.38%	1.3%	9%	6%	0.34%

**Table 4:** Location of abdominal tuberculosis.

Author	Duodenum	Jejunum	Ileum	Caecum	Ileocaecal	Appendix	Colon	Peritoneum	Mes_LYMPHNODES	Omentum	Gallbladder	Liver/Spleen
Akgun Y et al. [10]								37				
Yilmazlar et al. [11]		7	8					15				4
Barot et al. [12]			21		13		8					
mousa et al. [13]					3						1	
Chalya et al. [14]		12	72		122	6	6		10			7
Ali et al. [15]												
Wani et al. [16]			8	9		1		7	16	1		
Kita et al. [17]												
Rahman et al. [18]	1		3		22		8	8	4			
Klimach et al. [19].		1	21	5	18	2	8		11			2
Abbasi et al. [20].												1
Yousfani et al. [21]		2	17		21				1			
Ahmad QA et al. [22].		7	68									
Zhi Wang et al. [23]						2						
Mandavdhare et al. [24]			27	23	16		16					
Beloborodov et al. [25]		20	40	2		2	9		23			23
Singh et al. [26]		3	31		22		1	20	28			
Javed et al. [27]												
Iqbal et al. [28]			17	3	3				4			
Afridi SP et al. [29]		20	44		13		16					
Charokar et al. [30]		2	42	14			1	32	11			
Pathak et al. [31]		1	16		16	1	5					
Gondal S.H et al. [32]												
Cavalli et al. [33]												
Fillion et al. [34]			6					14	13	7		4
Chaudhary et al. [35]	4	30	260	16		16	6	276	247			11
Awasthi et al. [36]					39							
Kumar et al. [37]		1	4	1	10			6	6			
Mukhopadhyay et al. [38]												
Kim et al. [39]		4	14									
Gill P et al. [40]		1	2		3		2	9				4
Saaiq et al. [41]												
Sadiq et al. [42].												
Imran et al. [43]												
Arif et al. [44]												
Collado et al. [45]	2		7		6		4	1	8			2
Hassan et al. [46]	2		2					8	1			1
	9	111	730	73	327	30	90	433	383	8	1	59
	0.3%	4%	25%	2.5%	11%	1%	3%	15%	13%	0.27%	0.03%	2%

**Table 5:** Surgery performed; SMR_anastomosis: small bowel resection with primary anastomosis.

Author	Laparoscopy	Open	Conversion	Adhesiolysis	Stricturuplasty	Biopsy	Appendectomy	Lymphadenectomy	Small_Bowel_Resection with Ileostomy	SMR_Anastomosis	Direct_Suture	Hemicolectomy
Akgun Y et al. [10]	7	73	4			32				14		
Yilmazlar et al. [11]	0	17				10				5		
Barot et al. [12]	3	47		15	1	3			1	25		5
mousa et al. [13]	5	19	3			4			2	2		3
Chalya et al. [14]	NR	NR		124	1	8	6		1	56	12	14
Ali et al. [15].	0	6										
Wani et al. [16]	0	38		18	4	8	1	5		5		2
Kita et al. [17]	0	7								3		4
Rahman et al. [18]	0	4										
Klimach et al. [19]	4	72				16	13			6		22
Abbasi et al. [20].	0	10		6		1				2		
Yousfani et al. [21]	NR	NR		3	2	1			16	7	5	4
Ahmad QA et al. [22]	0	80			4	6			59	11		
Zhi Wang et al. [23]	NR	NR		15			2	13		9	9	
Mandavdhare et al. [24]	NR	NR										
Beloborodov et al. [25]	4	136	NR				2		22	31	22	
Singh et al. [26]	3	32	NR	2					21	12		2
Javed et al. [27]	0	30		11					12	5		2
Iqbal et al. [28]	0	40		15	5				17			3
Afridi SP et al. [29]	NR	NR		4					12		9	21
Charokar et al. [30]	6	66		22	4				9	10	13	14
Pathak et al. [31]	0	31			1		1		3	16		15
Gondal S.H et al. [32]	0	18			2	1			10		2	3
Cavalli et al. [33]	0	4										
Fillion et al. [34].	6	10										
Chaudhary et al. [35]	NR	NR		38	106					48	107	
Awasthi et al. [36]	NR	NR				11				34		
Kumar M. et al. [37].	1	41		2	3	1				8		10
Mukhopadhyay et al. [38]	0	64			4	6	7		19	6	2	17
Kim et al. [39]	0	18								13	5	
Gill P et al. [40]	4	7				4				3		
Saaiq et al. [41]	NR	NR		41	27	7			53	17		19
Sadiq et al. [42]	NR	NR		43	17				53			9
Imran et al. [43]	0	36		10	1				1	7	5	12
Arif et al. [44].	0	48		4	4	2			3	16		19
Collado et al. [45]	NR	NR				1						
Hassan et al. [46]	8	9				8		3		1		2

## Data Availability

The datasets analyzed during the current study are not publicly available but are available from the corresponding author on reasonable request.
